# Fluorine-Containing Ionogels with Stretchable, Solvent-Resistant, Wide Temperature Tolerance, and Transparent Properties for Ionic Conductors

**DOI:** 10.3390/polym16071013

**Published:** 2024-04-08

**Authors:** Xiaoxi Fan, Wenlong Feng, Shuang Wang, Yinpeng Chen, Wen Jiang Zheng, Jie Yan

**Affiliations:** School of Chemical Engineering, Sichuan University of Science and Engineering, Zigong 643000, China322086001116@stu.suse.edu.cn (Y.C.);

**Keywords:** ionogel, transparency, stretchability, temperature tolerance, solvent tolerance

## Abstract

Stretchable ionogels, as soft ion-conducting materials, have generated significant interest. However, the integration of multiple functions into a single ionogel, including temperature tolerance, self-adhesiveness, and stability in diverse environments, remains a challenge. In this study, a new class of fluorine-containing ionogels was synthesized through photo-initiated copolymerization of fluorinated hexafluorobutyl methacrylate and butyl acrylate in a fluorinated ionic liquid 1-butyl-3-methyl imidazolium bis (trifluoromethylsulfonyl) imide. The resulting ionogels demonstrate good stretchability with a fracture strain of ~1300%. Owing to the advantages of the fluorinated network and the ionic liquid, the ionogels show excellent stability in air and vacuum, as well as in various solvent media such as water, sodium chloride solution, and hexane. Additionally, the ionogels display impressive wide temperature tolerance, functioning effectively within a wide temperature range from −60 to 350 °C. Moreover, due to their adhesive properties, the ionogels can be easily attached to various substrates, including plastic, rubber, steel, and glass. Sensors made of these ionogels reliably respond to repetitive tensile-release motion and finger bending in both air and underwater. These findings suggest that the developed ionogels hold great promise for application in wearable devices.

## 1. Introduction

Ionogels consist of a cross-linked polymer network and a lot of ionic liquids (ILs) within it [1]. This combination leads them to behave macroscopically as elastomers and microscopically with the fluidity of ILs. As a result, ionogels, which are viscoelastic and ionically conductive, have been widely applied as solid electrolytes [2,3], energy harvesting devices [4,5,6], stretchable touch panels [7,8,9], skin-like sensors [10,11,12,13,14,15], electroactive actuators [16,17], and so on.

In practical applications, ionogels are usually designed in terms of composition and structure to give them properties that suit specific applications. Ideas for the preparation of ionogels are often borrowed from the preparation of hydrogels. For network construction, design ideas usually include dual network structures [18,19,20], interpenetrating networks [21,22,23], copolymer single-layer network structures [24,25,26], etc. The materials that make up the ionogel network are largely based on polyelectrolytes and hydrophilic polymers, such as polyvinyl alcohol [17,27,28], polyacrylic acid [29,30], and polyacrylamide [31,32]. Based on these strategies, ionogels have generally achieved high transparency and stretchability [8,33]. However, when used as flexible electronic devices such as sensors and actuators, the ionogels composed of hydrophilic networks often have unsatisfactory performance stability due to water absorption. 

Acrylate-based ionogels with high mechanical properties, stretchability, self-adhesiveness, and transparency have emerged as flexible electronic materials [34,35,36]. To enhance their stability in different solvent surroundings, fluorine-containing acrylate monomers and ionic liquids are used for constructing ionogels. Yue’s group has reported a series of copolymerized ionogels made of 2,2,2-trifluoroethyl acrylate and acrylamide monomers, which combine transparency, stretchability, solvent and temperature resistance, and high conductivity [37]. However, in addition to this, the study of fluorinated acrylate-based ionogels is still rarely reported. The preparation, performance study, and optimal design of fluorinated acrylate-based ionogels are still in their infancy.

Herein, we report the design and characterization of a class of ionogels made from fluorinated acrylate monomers and Ils that are highly stretchable (>1000%), transparent (>98%), and conductive (1.5 mS/cm in ambient). In addition, the ionogels are capable of maintaining a high conductivity of over 1.3 mS/cm at low temperatures, even down to −50 °C. They are also resistant to water (polar) and hexane (non-polar). This study provides a kind of multifunctional ionogel with excellent comprehensive performance for ionically conductive flexible materials.

## 2. Experimental Section

### 2.1. Preparation of the Ionogels

All of the solvents and chemicals were purchased from the Adamas (Adamas-beta^®^, Shanghai, China) and used as received without further purification. To prepare ionogels, the fluorine-containing monomer hexafluorobutyl methacrylate (HFBA) and fluorine-free monomer butyl acrylate (BA) (or ethyl acrylate (EA), pentyl acrylate (PA)) were dissolved in ionic liquid (IL) 1-butyl-3-methyl imidazolium bis (trifluoromethylsulfonyl) imide ([BMIM][NFSI]) along with photoinitiator 184 and crosslinker poly(ethylene glycol) diacrylate (average Mn 500) to form a transparent precursor. The gel fraction, the mass percentage of all solutes, ranged from 30% to 50%. The molar ratio between the fluorine-containing and fluorine-free monomers can be adjusted to 1:0, 7:3, 5:5, 3:7, and 0:1, where 1:0 and 0:1 denote pure PHFBA and PBA (or PEA) ionogel, respectively. The molar percentages of PEGDA and photoinitiator 184 to monomers were 0.4% and 0.5%, respectively. Then, the solution was poured into a glass mold with a 2 mm thickness of silicone rubber spacer in the center and cured for 10 h under 365 nm UV to obtain the ionogels.

### 2.2. Characterization of the Ionogels

The transparency tests were performed through a UV-Vis spectrophotometer (QFPR0580, Ocean Optics, Shanghai, China). The ionogels were prepared in the cuvette in situ for testing with a wavelength ranging from 400 to 760 nm with the reference of air.

### 2.3. Mechanical Tests

The mechanical tests were performed on an electronic tensile machine (AGX-5KNVD, SHIMADZU, Kyoto, Japen) with a 50 N load cell. For tensile tests, the ionogels were cut into a dumbbell shape, and the dimensions of the stenosis were 12 × 2 × 2 mm^3^. All tensile tests were conducted with a tensile speed of 100 mm/min. For adhesion tests, the ionogels were prepared into a rectangular shape with the dimensions of 100 × 20 × 2 mm^3^ for length× width× thickness. The specimens were adhered to a substrate (the plates such as glass, steel, copper, etc.) with the dimensions of contact surface of 20 × 20 mm^2^. A stiff PET film was adhered to the back of the ionogel to prevent elongation under the test. The adhesion strength was taken as the maximum stress under a uniaxial tensile speed of 100 mm/min. 

### 2.4. Contact Angle Measurement

The contact angle of water or hexane on ionogels was tested using the optical contact angle meter and interface tensiometer (USA KINO Industry Limited, Somerville, MA, USA). Briefly, a quantitative liquid was automatically extruded through a syringe and adhered to the surface of an ionic liquid gel, then a camera photographed the contact surface, and the contact angle was determined through software analysis.

### 2.5. Swelling Property of the Ionogels

The swelling property of ionogels in different solvents was tested using the mass method. First, the original mass of the gel was weighed and denoted as *m*_0_. Then, it was immersed in the solvent (such as NaCl solution, and hexane), and once the mass remained constant, it was measured again and denoted as *m*_1_. The swelling ratio of the ionogel was calculated as (*m*_1_/*m*_0_) × 100%.

### 2.6. Thermal Analysis

Differential Scanning Calorimetry (DSC) experiments were measured by a Netzsch DSC200F3 analyst (Selb, Germany) at a cooling and heating speed of 10 °C/min, and the TGA measurements were performed on a Netzsch STA409PC (Selb, Germany) integrated thermal analyst via scanning a temperature ranging from 25 to 600 °C with a heating speed of 10 °C/min. The gas medium for thermal analysis was nitrogen.

### 2.7. Impedance Tests

The impedance of the ionogels was tested by an impedance analyst (6632, SAIMR, Suzhou, China) using a solid-state dielectric fixture (FX-000C20). The ionogel samples were cut into a cylinder with a radius of 25 mm (*r*) and height of 2 mm (*L*), and placed in the middle of the fixture to obtain the resistance *R*_b_. The conductivity (*σ*) can be calculated by the equation below:

*σ* = *L*/(*R*_b_ × π × *r*^2^)

### 2.8. Cyclic Tensile Load-Unload Test

The cyclic tensile load-unload test was conducted using an electron universal testing machine (CMT6503, SANS, Shenzhen, China). In brief, the ionogel sample was clamped between two fixtures, maintaining a flat and relaxed state with zero strain. The program was then initiated to stretch the sample to 110% of its original length at a rate of 100 mm/min. Subsequently, the sample was retracted back to 90% at the same rate. This cyclic process was repeated, with the computer recording the stress changes during the procedure.

### 2.9. Fabricating of the Ionogel-Based Sensors

The ionogel was cut into a shape of 600 × 10 × 2 mm^3^. Two copper electrodes were attached at the two ends of the ionogel on the upper and lower surfaces, respectively. The silicon membrane was used to cover the ends for insulation.

## 3. Results and Discussion

**Scheme 1:**
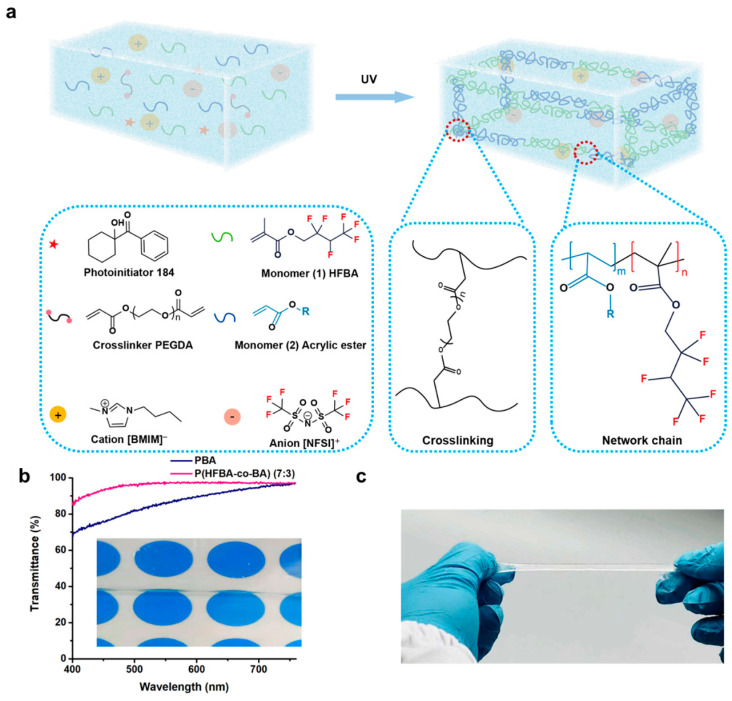
Preparation and display of the ionogels. (**a**) Schematic illustration of the one-step preparation process of the ionogels, HFBA and acrylic ester monomers are copolymerized in IL under irradiation of 365 nm UV, and simultaneously crosslinked by PEGDA to obtain ionogels; (**b**) transmittance spectrum of ionogels P(HFBA-*co*-BA) and pure PBA with a film thickness of 2 mm. Inset: photograph of the ionogel with a thickness of 2 mm over an image with a polka dot pattern; (**c**) demonstration of the elasticity of the ionogel.

As depicted in Scheme 1a, the ionogels were prepared using a one-step photoinitiated copolymerization approach where hexafluorobutyl methacrylate (HFBA) was combined with acrylic ester (butyl acrylate (BA) or ethyl acrylate (EA)) in ionic liquid (IL), along with poly (ethylene glycol) diacrylate (PEGDA) as a crosslinker. HFBA, a fluorinated monomer with multiple fluorine atoms, was chosen to form the fluoropolymer network in the ionogels, thereby enhancing their solvent resistance. In addition, EA and BA were used as copolymers with HFBA to improve the mechanical and adhesion properties. The fluorine-containing IL 1-butyl-3-methyl imidazolium bis (trifluoromethylsulfonyl) imide ([BMIM][NFSI]) provides excellent solvent resistance and low-temperature tolerance, and imparting the ionogels good stability in different application scenarios. The resultant fluorine-containing copolymer ionogel shows excellent transparency, as Scheme 1b shows, P(HFBA-*co*-BA) has an average transmittance of over 95% in the visible range of 400 to 760 nm. While the pure PBA ionogel only has 85% of the average transmittance, this indicates that the fluorinated components can improve the transparency of the ionogel. In addition, the copolymer ionogel has good stretchability (Scheme 1c), which can accommodate the need for large deformation responses in sensors and actuators.

**Figure 1:**
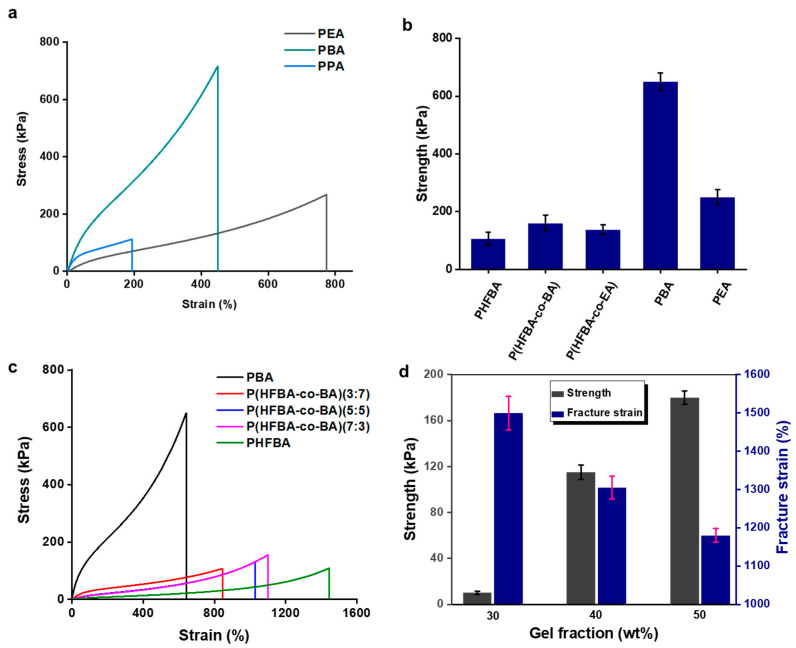
Mechanical properties of the ionogels. (**a**) Tensile stress-strain curves of PEA, PBA, and PPA ionogels (gel fraction is 50%); (**b**) tensile strength of five kinds of ionogels (gel fraction is 50%) including pure PHFBA, PBA, PEA ionogels, and copolymerized P(HFBA-*co*-BA), P(HFBA-*co*-EA) ionogels; (**c**) tensile stress-strain curves of P(HFBA-*co*-BA) ionogels (gel fraction is 50%) with different molar ratio between HFBA and BA of 1:0, 7:3, 5:5, 3:7, 0:1, where 1:0 and 0:1 represent pure PHFBA and PBA ionogels respectively; (**d**) strength and fracture strain of P(HFBA-*co*-BA) (7:3) ionogels with gel fraction of 30%, 40% and 50%. All samples are cut into dumbbell shapes with a 12 mm length of test section, and the tensile speed is 100 mm/min.

The polyacrylate-based ionogels in this work were prepared using various monomers, including HFPA, BA, EA, and PA both individually and in combination. Figure 1a shows the results of the tensile tests of the pure PEA, PBA, and PPA ionogels. Among them, the PBA ionogel has the highest strength, while the PEA shows the best ductility. By copolymerizing EA or BA with HFBA, the resultant copolymer ionogels P(HFBA-*co*-BA) and P(HFBA-*co*-EA) have higher strength than pure PHFBA ionogel (Figure 1b). It is noteworthy that the ionogels composed solely of PBA and PEA exhibit considerably higher tensile strength than the other groups. This suggests that the fluorine-free chain segments, namely the PBA and PEA segments, contribute to the strength of copolymer ionogels.

To verify this view, P(HFBA-*co*-BA) ionogel with stronger strength compared with P(HFBA-*co*-EA) is selected for further investigation. By altering the molar ratio between HFBA and BA, a series of P(HFBA-*co*-BA) (x:y) copolymer ionogels were prepared for the tensile test, where x and y represent the proportions of HFBA and BA, respectively. Figure 1c shows that the fracture strain increases as the HFBA molar ratio increases, while the strength of the ionogels exhibits the opposite trend. This may be related to the affinity of the molecular chains composed of the two monomers to the IL. The ionogel containing more fluorinated segments have a higher affinity for fluorine-containing IL and are more flexible, thus contributing to the ductility of ionogels. Conversely, the non-fluorinated chain segments are more rigid, which favors strength. By optimizing, P(HFBA-*co*-BA) (7:3) exhibits the best strength (182.5 kPa of fracture strength) and ductility (1170.9% of fracture strain) among all copolymer ionogels. The effect of gel fraction on mechanical properties has also been studied. Figure 1d shows the mechanical properties of P(HFBA-*co*-BA) (7:3) ionogels with varying gel fractions. With the increase of gel fraction from 30% to 50%, the strength increased significantly from 10.1 kPa to 180.2 kPa, while the fracture strain decreased from 1507.0% to 1180.5%.

**Figure 2:**
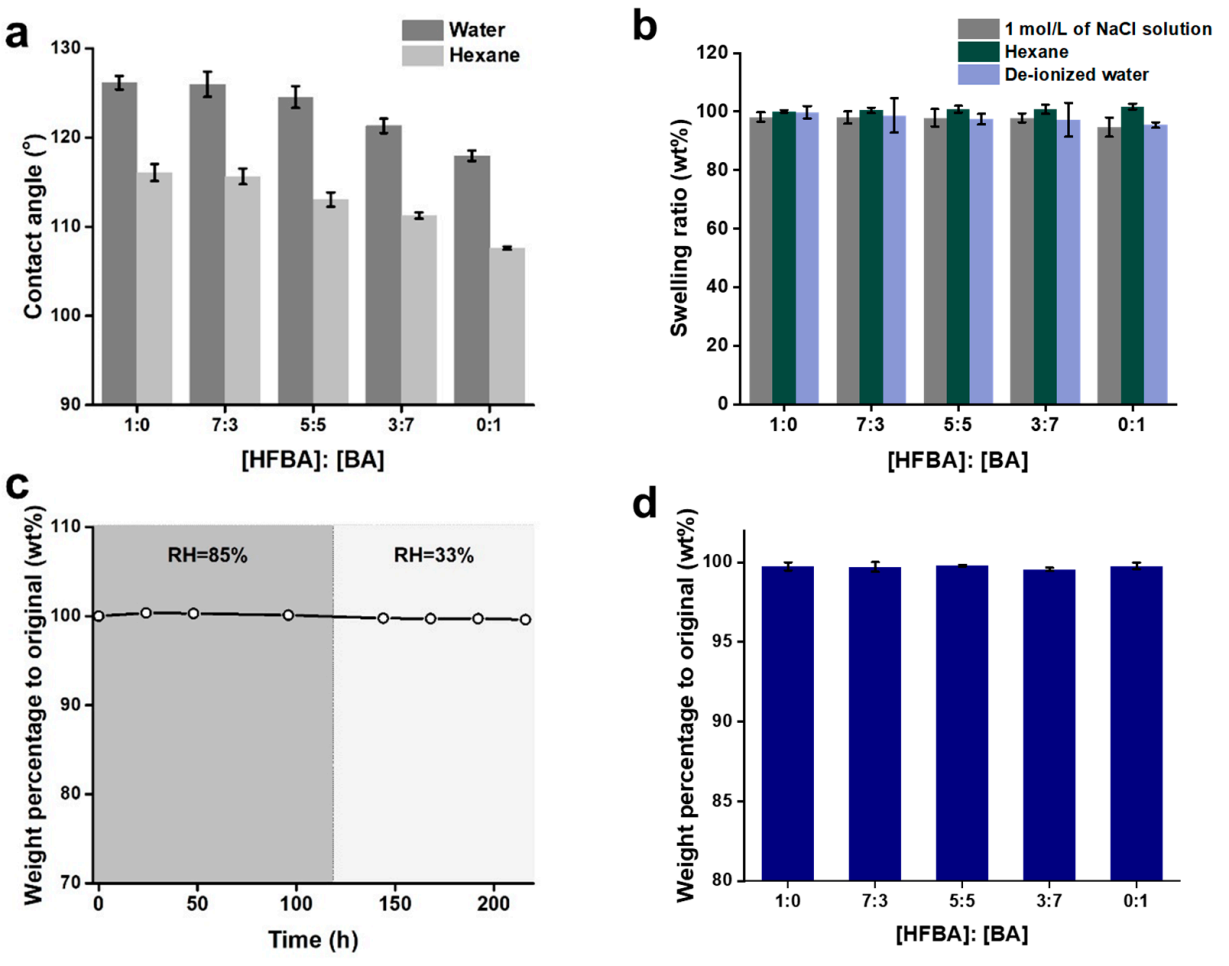
Stability of ionogels under different environments. (**a**) Contact angle of deionized water and hexane on P(HFBA-*co*-BA) ionogels; (**b**) swelling ratio of P(HFBA-*co*-BA) ionogels in NaCl solution, de-ionized water and hexane; (**c**) mass stability of P(HFBA-*co*-BA) ionogels in air with relative humidity of 85% and 33%; (**d**) mass stability of P(HFBA-*co*-BA) ionogels in vacuum for 24 h.

The solvent resistance of P(HFBA-*co*-BA) ionogels is assessed by examining the surface contact angles of polar water and non-polar hexane, as depicted in Figure 2a. In the case of the ionogel comprising solely HFBA, the contact angles for water and hexane are measured at 127.2° and 116.5°, respectively. Contacting angle of P(HFBA-*co*-BA) (7:3) is comparable to that of the pure PHFBA. Subsequently, as the fluorine content decreases, the contact angle diminishes significantly. The ionogel composed solely of PBA has contact angles of 119.7° for water and 98.4° for hexane. This result suggests that the PHFBA chains containing fluorine have a beneficial effect in repelling both polar and non-polar reagents.

To investigate the effect of network composition on the stability of ionogel in solvents, the equilibrium swelling ratios of ionogels in NaCl water solution, de-ionized water, and hexane are determined using the mass method. Ionogels of P(HFBA-*co*-BA) with BA samples with weights ranging from 1:0 to 0:1 are prepared and their original weight is recorded as *m*_0_. The samples are then immersed in solvent until their weight stabilizes, and the equilibrium weight are recorded as m_e_. The swelling ratio is determined as the percentage of *m*_0_ over *m*_e_. Figure 2b shows that as the fluorine component increases, the swelling ratio in NaCl solution, de-ionized water, and hexane approaches 100%, signifying greater stability. The copolymer P(HFBA-*co*-BA) (7:3) ionogel exhibits equivalent stability as pure PHFBA. Figure 2c shows the weight change P(HFBA-*co*-BA) (7:3) ionogel over time at different relative humidity (RH) levels. The weight of all ionogels remained stable when exposed to 85% and 33% RH for over 100 h. Additionally, due to the non-volatility of ionic liquids, the ionogels experienced minimal mass loss under vacuum conditions, with the weight percentage remaining above 99% of the original mass after 24 h, as shown in Figure 2d. These results collectively indicate that the polyacrylate-based ionogels exhibit stability in various solvent, atmospheric, and vacuum conditions.

**Figure 3:**
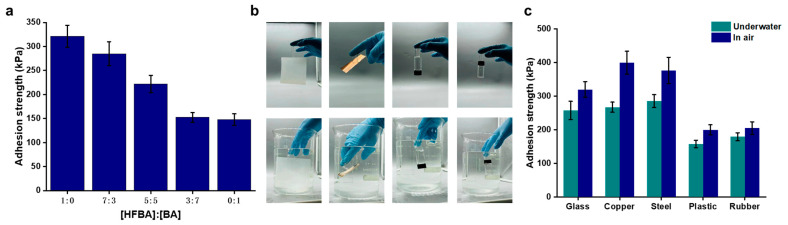
Adhesion properties of P(HFBA-*co*-BA) ionogels. (**a**) Molar ratio of HFBA to BA on adhesion strength of P(HFBA-*co*-BA) ionogels to steel plates; (**b**) adhesion demonstrations in air and underwater of ionogels on various substrates, such as silicone rubber, copper foil, glass bottle, and plastic cap; (**c**) adhesion strength of P(HFBA-*co*-BA) (7:3) ionogels on different substrates.

Since the ionogels have excellent water resistance performance, their adhesion behavior with various substrates underwater has been studied. Figure 3a shows that the ionogel with higher fluorine content has better hydrophobicity (Figure 2a), resulting in higher adhesion strength to steel plates. Among all copolymer ionogels, P(HFBA-*co*-BA) (7:3) exhibits the largest adhesion strength of 284.8 kPa, which is comparable to pure PHFBA. Figure 3b illustrates the adhesion phenomenon of P(HFBA-*co*-BA) (7:3) ionogel to different substrates including silicone rubber, copper foil, glass, and plastic in both air and water. The ionogel exhibits good adhesion to various substrates in different environments. Figure 3c shows the adhesion strength of P(HFBA-*co*-BA) (7:3) to different substrates in air and underwater. In comparison to the test results conducted in the air, it is observed that the adhesion energy of the ionogel diminishes when submerged underwater. However, the adhesion strengths for all substrates exceed 150 kPa. This is due to the excellent hydrophobicity of the PHFBA component. The adhesion strength is larger when adhering to steel, copper, and glass compared to plastic and rubber. The reason for this is that substrates such as steel, copper, and glass have higher surface energy than plastic and rubber. As a result, the polymer chains of the ionogel can more easily infiltrate the microstructure of the substrate surface, resulting in stronger adhesion. These results indicate that the fluorine component can improve the adhesion property of ionogel underwater.

**Figure 4:**
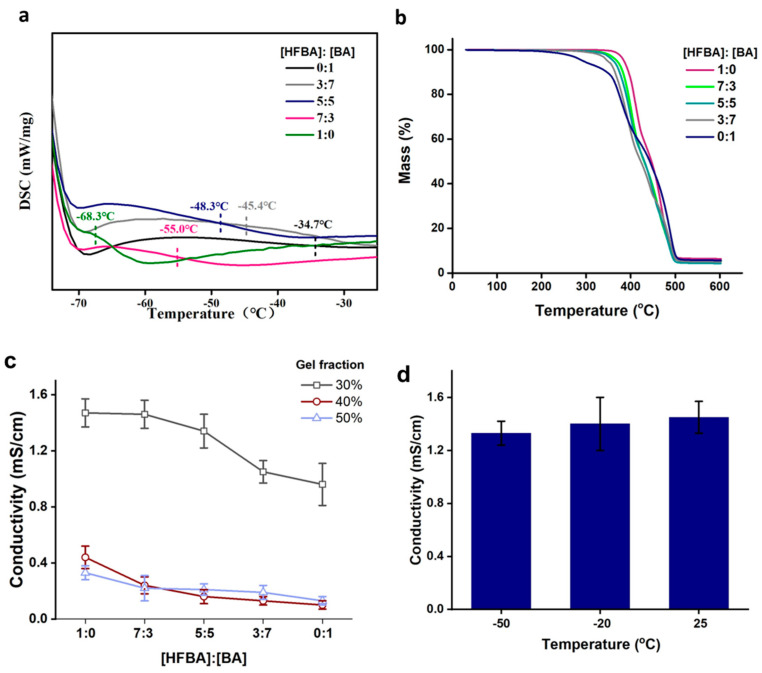
Thermal stability and conductivity of ionogels. (**a**) The TG results of the ionogels made of different molar ratios between HFBA and BA; (**b**) the DSC curves of the ionogels; (**c**) Conductivity of ionogels with gel fraction ranging from 30% to 50%; (**d**) the temperature-dependent ionic conductivity of the P(HFBA-*co*-BA) (7:3) ionogel with gel fraction of 30%.

To investigate the low-temperature resilience of the ionogels, we conducted differential scanning calorimetry (DSC) to determine the glass transition temperature (Tg) of the specimens (Figure 4a). The samples tested included pure PBA ([HFBA]:[BA] = 0:1), pure PHFBA ([HFBA]:[BA] = 1:0), and three ionogel samples with varying molar ratios of HFBA to BA. The pure PBA ionogel exhibits the highest Tg at −48.3 °C. As the molar ratio of HFBA increases, the Tg of the ionogels decreases. When the ionogel consisted entirely of PHFBA, the Tg reaches its lowest value at −68.3 °C. This reduction in Tg can be attributed to the fluorine-containing side groups of PHFBA, which reduce polarity, resulting in decreased internal rotation activation energy and intermolecular forces.

We also studied the high-temperature resilience of the ionogels by analyzing the thermal decomposition using thermal gravimetric analysis (TGA) in a nitrogen environment (Figure 4b). The pure PBA ionogel begins to decompose at approximately 200 °C. Introducing a fluorine component in the ionogel led to an increase in the decomposition temperature of the PHFBA/PBA ionogel as the molar ratio of PHFBA content increased. The pure PHFBA ionogel exhibits the highest decomposition temperature at around 350 °C due to the high thermal stability of the fluorine side groups in the PHFBA chains, which retard the thermal decomposition of the ionogels. These results indicate that the fluorine ionogels, with good thermal stability, can be used in temperatures ranging from −60 °C to 350 °C.

The electrical performance of ionogels as an ionic conductor was investigated. Figure 4c shows the conductivity of ionogels with varying molar ratios of HFBA to BA and gel fraction. As the molar ratio of HFBA increases, the conductivity of the ionogels shows a rising trend. Specifically, for ionogels with a 30% gel fraction, the conductivity significantly increases from 0.94 mS/cm (HFBA:BA = 0:1) to 1.42 mS/cm (HFBA:BA = 7:3), and then remained stable till HFBA:BA = 1:0. This can be attributed to the low polarity of the fluorine-containing PHFBA chains, which reduces the physical interaction of the gel network with the ionic liquid molecules, allowing for freer movement through the gel network and thus increasing the conductivity. Furthermore, with the increasing gel fraction, the conductivity of the ionogels decreases significantly. For P(HFBA-*co*-BA) (7:3) ionogel, its conductivity decreases from 1.44 mS/cm to 0.21 mS/cm as the gel fractions increase from 30% to 50%.

The PHFBA/BA (7:3) ionogel also maintains stable electrical performance over a wide temperature range. The conductivity only slightly decreases from 1.41 mS/cm to 1.35 mS/cm as the temperature drops from 25 °C to −50 °C (Figure 4d). The fluorine-containing ionogel’s excellent low-temperature tolerance (Figure 4a) is attributed to its highly elastic gel network and does not impede the mobility of the ionic liquid inside even at −50 °C.

**Figure 5:**
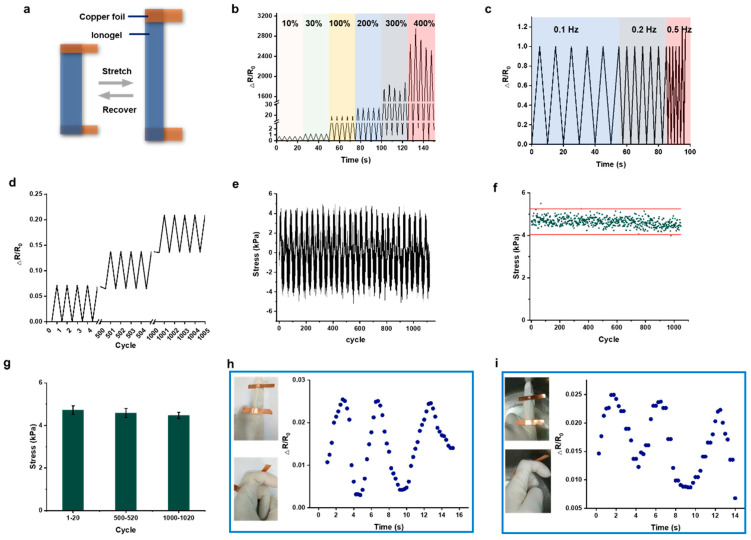
Electrical and sensing properties of the P(HFBA-*co*-BA) (7:3) ionogel. (**a**) Schematics of the ionic conductor; (**b**) relative resistance of the ionic conductor under different strains ranging from 10% to 400%; (**c**) real-time recording of relative resistance variation of the ionic conductor under different tensile-release frequencies ranging from 0.5 Hz to 1 Hz; (**d**) relative resistance response under tensile-release processes with a strain of 10% for over 1000 cycles; (**e**) real-time recording of the stress of the ionogel gel under recycle tensile-release processes with a strain of 10%; (**f**) the maximum stress value measured during each tensile-release cycle, the two red lines in the graph are parallel to the x-axis, and the data points are gradually shifted downwards as the number of loops increases; (**g**) average of the maximum stress values in different cycling phases, including cycle 1 to 20, 500 to 520, as well as 1000 to 1020; (**h**) signals of relative resistance during finger bending in air; (**i**) relative resistance change during finger bending underwater.

The sensing properties during cyclic tensile-release are investigated using copper foil connected to both sides of the P(HFBA-*co*-BA) ionogel as shown in Figure 5a. The ionogel-based sensors under low strain (10–30%) and high strain (100–400%) shows reversible resistance responses (Figure 5b). The changes in relative resistance remain stable throughout the repeated tests at 30% strain with a tensile-release frequency between 0.1 and 0.5 Hz (Figure 5c). For over 1000 cycles, the relative resistance value gradually drifts towards higher values, but the difference between the very high and very low values remains constant (Figure 5d). This is because the ionogel develops irreversible deformation after a lot of tensile-release cycles. To end this, cyclic load-unload testing has been conducted using a fatigue testing machine (Figure 5e). After ~1000 cycles of loading-unloading, the maximum stress values at each cycle shown in Figure 5f reveals a downward trend. We take the maximum values of the 1–20, 500–520, and 1000–1020 cycles respectively for averaging, and the results are shown in Figure 5g. The maximum stress decreases with the increase of the cycle. This means the ionogel undergoes irreversible elongation during repeated loading-unloading processes. This will further lead to an increase in resistance as Figure 5d shows. Even though, the ionogel-based sensor still responds to deformation with a stable signal change. 

Based on these results, the P(HFBA-*co*-BA) (7:3) ionogel is used to detect human activity. Its adhesive properties facilitated the direct attachment of the sensor. As shown in Figure 5h, the strip sensor is attached to the finger joint to monitor the movement of the finger. When the finger is bent from 0° to 90°, the change in the relative resistance of the sensor increases accordingly, and the electrical signals can be repeated. More importantly, when the sensor is used underwater, the electrical signals can still change with the movement of the finger (Figure 5i). This indicates that the ionogel can be used in a water environment.

## 4. Conclusions

In summary, a class of fluorinated ionogels is developed by a simple one-step photoinitiated copolymerization of HFBA and BA in a hydrophobic ionic liquid [BMIM][NFSI]. The fluorine-containing PHFBA segments provide the ionogels with excellent transparency, stretchability, hydrophobicity, adhesiveness, and temperature tolerance; while the PBA segments impart strength to the ionogel network. Under the synergistic effect of fluorinated IL, the resultant ionogels exhibit good stability in air and vacuum environments, as well as in various liquid media such as water, NaCl solution, and hexane. The ionogel-based sensors are successfully exploited to detect the response behaviors to recycle tensile-release and finger-bending motion. The response signal maintains good stability over 1000 cycles and in environments such as in the air or underwater. The ionogels reported in this work have great potential for the development of wearable devices with solvent-resistant, self-adhesive performance.

## Data Availability

The data presented in this study are available on request from the corresponding author (due to privacy).
